# Antiphagocytic protein 1 increases the susceptibility of *Cryptococcus neoformans* to amphotericin B and fluconazole

**DOI:** 10.1371/journal.pone.0225701

**Published:** 2019-12-04

**Authors:** Muhammad Ghaffar, Cody Orr, Ginny Webb

**Affiliations:** Division of Natural Sciences and Engineering, University of South Carolina Upstate, Spartanburg, South Carolina, United States of America; University of Minnesota, UNITED STATES

## Abstract

*Cryptococcus neoformans* is a facultative intracellular pathogen responsible for the most common cause of fungal meningioencephalitis, occurring primarily in immunocompromised individuals. Antiphagocytic protein 1 (App1) is a virulence factor produced by *C*. *neoformans* that inhibits phagocytosis of the yeast by host macrophages. Treatment of cryptococcosis includes amphotericin B, fluconazole, and flucytosine. Virulence factors have been shown to affect the susceptibility of the pathogen to antifungal drugs. In this study, we aimed to examine the relationship between App1 and antifungal drugs. We found that short-term exposure to amphotericin B downregulates *APP1* expression while exposure to fluconazole upregulates *APP1*. In addition, App1 was found to increase the susceptibility of the yeast to amphotericin B and fluconazole. This study provides evidence of an intricate relationship between App1 and antifungal drugs.

## Introduction

*Cryptococcus neoformans* is an environmental fungal pathogen that is the leading cause of fungal meningitis resulting in approximately 278,000 cases and 181,000 deaths each year. This disease accounts for approximately 15% of AIDS-related deaths worldwide [1–3]. *C*. *neoformans* is an opportunistic, facultative intracellular pathogen and cryptococcal meningitis has risen as a major cause of illness and mortality due to the growing population of immunocompromised individuals [4]. Rates of cryptococcal meningioencephalitis are highest in 3 risk groups: (1) HIV infected individuals, (2) organ transplant recipients, (3) and chemotherapy recipients [5].

Infection begins with inhalation of spores or desiccated yeast cells into the host lung. The pathogen can be efficiently cleared and contained in an immunocompetent host through an immune response including phagocytosis of *C*. *neoformans* by alveolar macrophages. However, in an immunocompromised host with an inadequate immune response, the yeast can thrive and avoid destruction by the host. The organism will then grow and disseminate through the blood to the central nervous system resulting in meningioencephalitis [6].

If left untreated, cryptococcal meningoencephalitis can be fatal. There are three primary antifungal drugs involved in the lengthy treatment of cryptococcosis. Initial therapy includes amphotericin B (AMB) and flucytosine (5FC) for at least two weeks followed by fluconazole (FLC) for eight weeks [7]. AMB is a polyene that binds to ergosterol in the fungal membrane allowing the leakage of fungal cell content (5). FLC inhibits the production of ergosterol in the fungal membrane leading to disruption of the membrane and the build up of toxic sterols [8]. Flucytosine is converted to fluorouracil within the yeast cell, which inhibits DNA synthesis [9, 10].

*C*. *neoformans* expresses various virulence factors to aid its ability to overtake the host immune response. These virulence factors include a capsule, melanin production, and the ability to grow at 37°C. Another important virulence factor of *C*. *neoformans* is Antiphagocytic Protein 1 (App1), which inhibits alveolar macrophages from phagocytizing the yeast cell [11–14]. In previous studies, it has been shown that environmental conditions that resemble the mammalian lung (i.e. low glucose concentration, 5% CO_2_, and 37°C) result in upregulation of *APP1* [15]. The lung plays a critical role in disease development, as this is where the initial interaction between *C*. *neoformans* and the host’s macrophages takes place. An upregulation of *APP1* in this environment can therefore have a significant effect on disease outcome. In an immunocompetent host, the inhibition of phagocytosis by App1 allows the yeast to go undetected, grow, and eventually disseminate [12]. However, in an immunocompromised host, inhibition of phagocytosis of the yeast can actually be beneficial for the host. This is due to the fact that when *C*. *neoformans* is phagocytized in an immunocompromised host it is not efficiently killed inside the macrophage and has been shown to grow faster intracellularly, leading to faster disease progression. Therefore, infection by *Δapp1* in an immunocompromised mouse model results in a hypervirulent infection. The expression of *APP1* can therefore have different effects on disease progression dependent on the immune state of the host [12].

Previous studies have shown that antifungal drugs regulate gene expression and virulence factors of *C*. *neoformans* [16, 17]. In addition, studies have also shown that virulence factors of *C*. *neoformans* have been found to affect the susceptibility of the yeast to the commonly used antifungal drugs [18–20]. Due to the role that App1 plays in the virulence of *C*. *neoformans*, we aimed to investigate the effect of antifungal drugs on the expression of *APP1* as well as the effect of App1 on the susceptibility of *C*. *neoformans* to antifungal drugs. The data presented here, suggest that *APP1* is affected by both AMB and FLC in opposite directions, with AMB downregulating *APP1* expression and FLC upregulating *APP1* expression. In addition, data show that App1 increases the susceptibility of *C*. *neoformans* to both AMB and FLC.

## Materials and methods

### Strains, media, and reagents

*C*. *neoformans* var. *grubii* serotype A strain H99 was the wild-type (WT) strain and the *Δapp1* strain was used for comparisons. The *Δapp1*^*Rec*^ strain was used as a control strain. In this strain, the *APP1* gene was reintroduced into the *Δapp1* strain. We used this strain to ensure any observed differences between the WT and *Δapp1* strains are due to the absence of App1 and are not artifacts of the production of the *Δapp1* strain. These strains have been previously used to study App1 [12, 15]. All strains were kindly provided by Dr. Maurizio Del Poeta, Stony Brook University. *C*. *neoformans* cells were grown at 30°C in yeast-peptone-dextrose (YPD) media. The antifungal drugs AMB and FLC were obtained from Fisher Scientific and Sigma respectively.

### Effect of antifungal drugs on *APP1* expression

*C*. *neoformans* cells were grown for 18–20 hours at 30°C while shaking. Cells were centrifuged and washed three times using sterile phosphate-buffered saline (PBS). 2 x 10^7^ cells/ml of H99 (WT) or *Δapp1* cells were incubated with varying doses of AMB (0.125, 0.25, and 0.5 μg/mL) or FLC (8, 16, and 32 μg/mL) in an automated shaker for 90 minutes at 30 °C. Killing assays revealed no effect on *C*. *neoformans* survival when 2 x 10^7^ cells/ml were treated with these antifungal concentrations for the 90-minute time period, suggesting that any change in mRNA levels is not due to cell death. The cells were then lysed and RNA was extracted and used to measure *APP1* gene expression as described below.

### RNA extraction and real-time PCR

A RNeasy minikit (Qiagen, Valencia, CA) was used to extract RNA from cells. Acid-washed glass beads were used to homogenize the cells to begin RNA extraction. The extraction process was then completed using the kit. RNA (1ug) was converted to complementary DNA using a SuperScript first-strand synthesis reverse transcription kit (Invitrogen, Carlsbad, CA). Real-time PCR was performed to measure *APP1* mRNA expression. A 25μl mix was made using cDNA, SYBR green, 1μl each of 10μM reverse and forward primers, and H_2_O. A Bio-Rad CFX Connect Real Time detection system was used for the real time PCR. Previously established reaction cycle conditions were used [11]: 95°C for 3 min for denaturation, 40 cycles of 95°C for 10 sec for denaturation and 58°C for 45 sec for annealing, 95°C for 1 min, and 58°C for 1 min. A melting curve was then completed starting at 58°C for 1 min intervals, increasing 0.5°C each cycle for 78 cycles. The average cycle threshold was given for each sample and normalized to the control gene Actin. Data was analyzed using the CFX Manager software (BioRad, Hercules, CA). All reactions were carried out in triplicate. Primers used were: App1F (5_-GAC GAT GAG TTG GAG GAA CC-3_), App1R (5_-CGA GAG CAG CCT CAA TGA C-3_), ACTF (5_-CTG TCT TCC CTT CTA TTG TTG GTC-3_), and ACTR (5_-CTC AAT GGG GTA CTT CAA GGT AAG-3_).

### Effect of antifungal drugs on growth of *C*. *neoformans*

Cultures of *C*. *neoformans* (WT and *Δapp1*) were grown for 18–20 hours in YPD and washed three times with sterile PBS and counted. One ml of yeast nitrogen base (YNB) media containing 10^5^ cells was plated into a 12 well plate. The antifungal drugs, FLC (3μg/ml) and AMB (0.4μg/ml) were added into the appropriate wells. Control wells were treated with the same volume of solvent, sterile H_2_0 (for AMB control) or DMSO (for FLC control). Plates were incubated at 30°C for 46 hours in a BioTek plate reader while shaking. The OD540nm was read every 2 hours to generate growth curves.

### Killing assay

H99 and *Δapp1* cells were incubated at a concentration of 2 x 10^3^ cells/ml with varying doses of AMB (0, 0.0625, 0.125, 0.25 μg/mL) or FLC (0, 0.5, 2.5, 5 μg/mL) in YPD media for 90 minutes at 30 °C. Following the incubation with antifungal drugs, cells were plated on YPD agar using serial dilutions. CFU were counted after incubation at 30 °C for 48 hours to measure the percent survival of H99 and *Δapp1* strains after treatment with AMB and FLC. Survival was measured by calculating the % survival of treated cells compared to non-treated cells. (CFU of cells treated with AMB or FLC/CFU of untreated cells x 100).

## Results

### Effect of antifungal drugs on *APP1* expression

**Fig 1:**
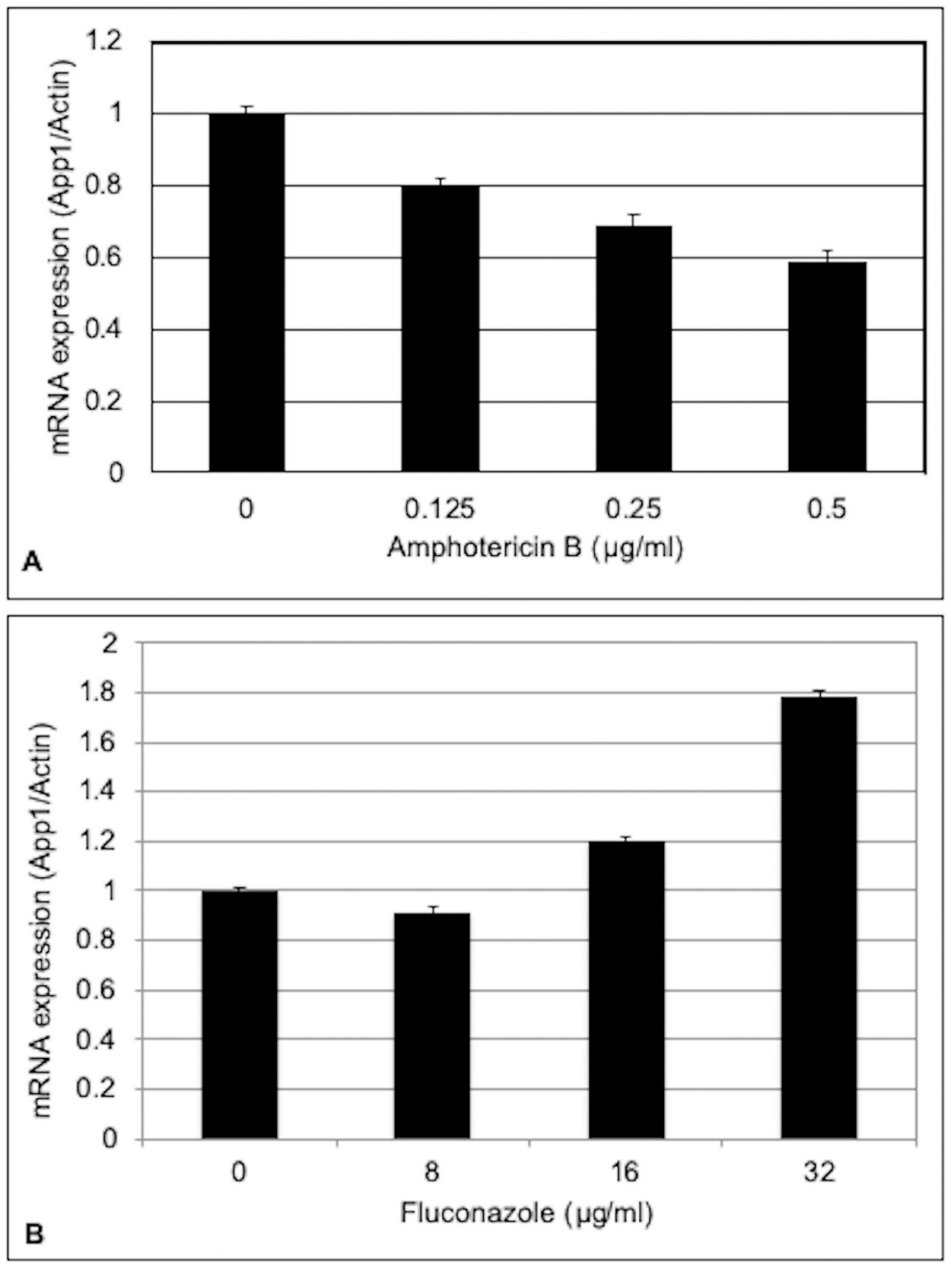
Antifungal drugs affect *APP1* expression. *C*. *neoformans* (WT or *Δapp1*) was grown in YPD media with amphotericin B (A) or fluconazole (B) for 90 minutes and then *APP1* expression was measured using qPCR. *APP1* expression is downregulated in response to amphotericin B and upregulated in response to fluconazole. Data is representative of three independent experiments.

In order to determine whether *APP1* was affected by AMB and FLC, cell cultures were exposed to the drug for 90 minutes and then *APP1* gene expression was measured using real time PCR. Killing assays were performed to ensure there was no killing of the cells at this concentration for this time period, therefore, any changes in gene expression are not due to cell death. Fig 1 presents the gene expression results of *APP1* expression following treatment with increasing doses of AMB or FLC. Fig 1a shows that short-term exposure to AMB downregulates the expression of *APP1* in a dose-dependent manner, with the highest dose of 0.5 μg/mL having the biggest effect on *APP1* expression. Alternatively, Fig 1b shows that FLC upregulates *APP1* expression after short-term exposure, most notably seen at a FLC concentration of 32 μg/mL. Expression data is representative of three individual trials each done in triplicate.

### App1 decreases growth of *C neoformans* following exposure to amphotericin B

**Fig 2:**
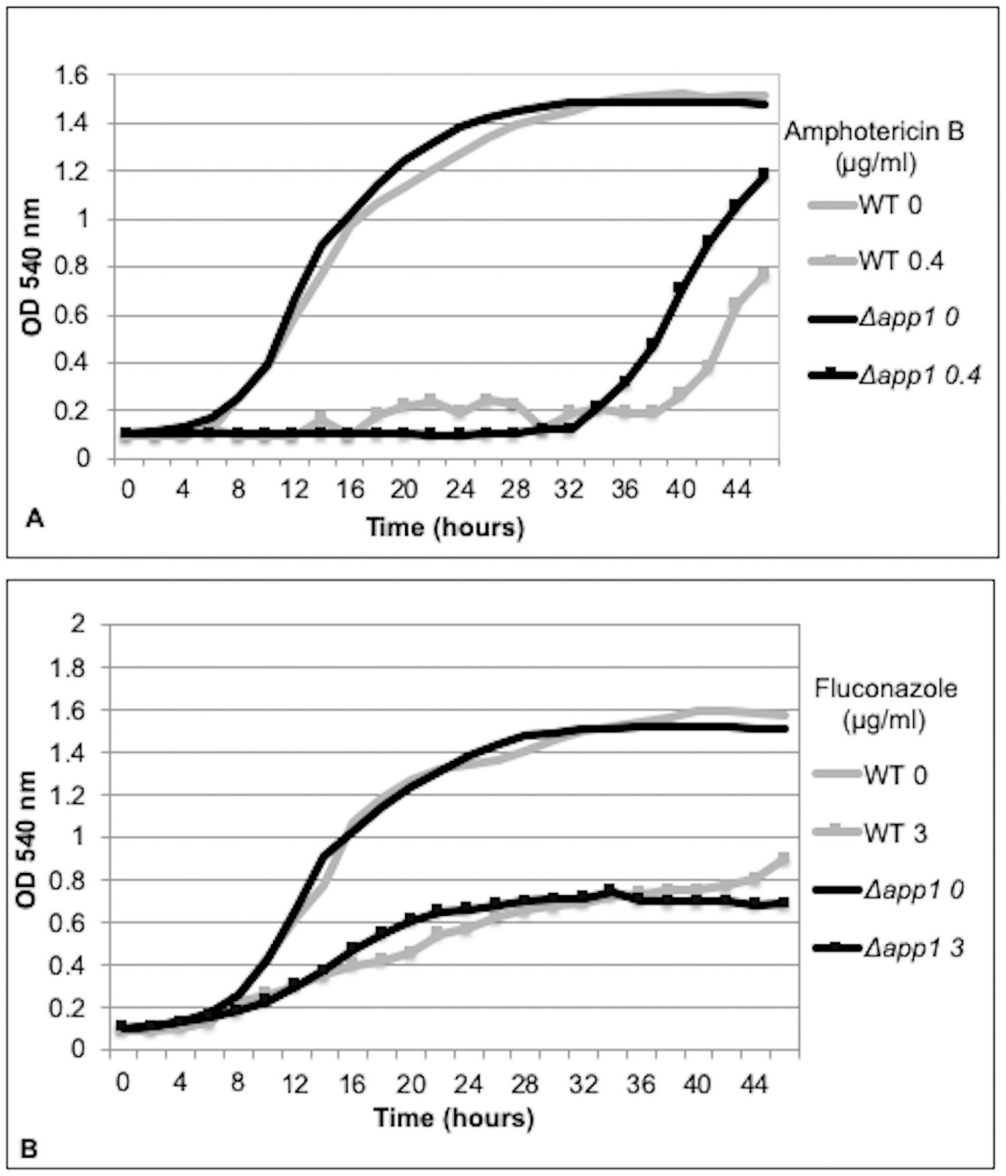
App1 decreases the recovery time after amphotericin B treatment. 10^5^ *C*. *neoformans* cells were treated with amphotericin B (A) or fluconazole (B) and their growth curves were graphed by measuring the OD 540nm for 46 hours while shaking. App1 increases the lag phase of cells treated with amphotericin B but had no effect on the growth curve following fluconazole treatment. Data are representative of at least 4 independent trials.

**Fig 3:**
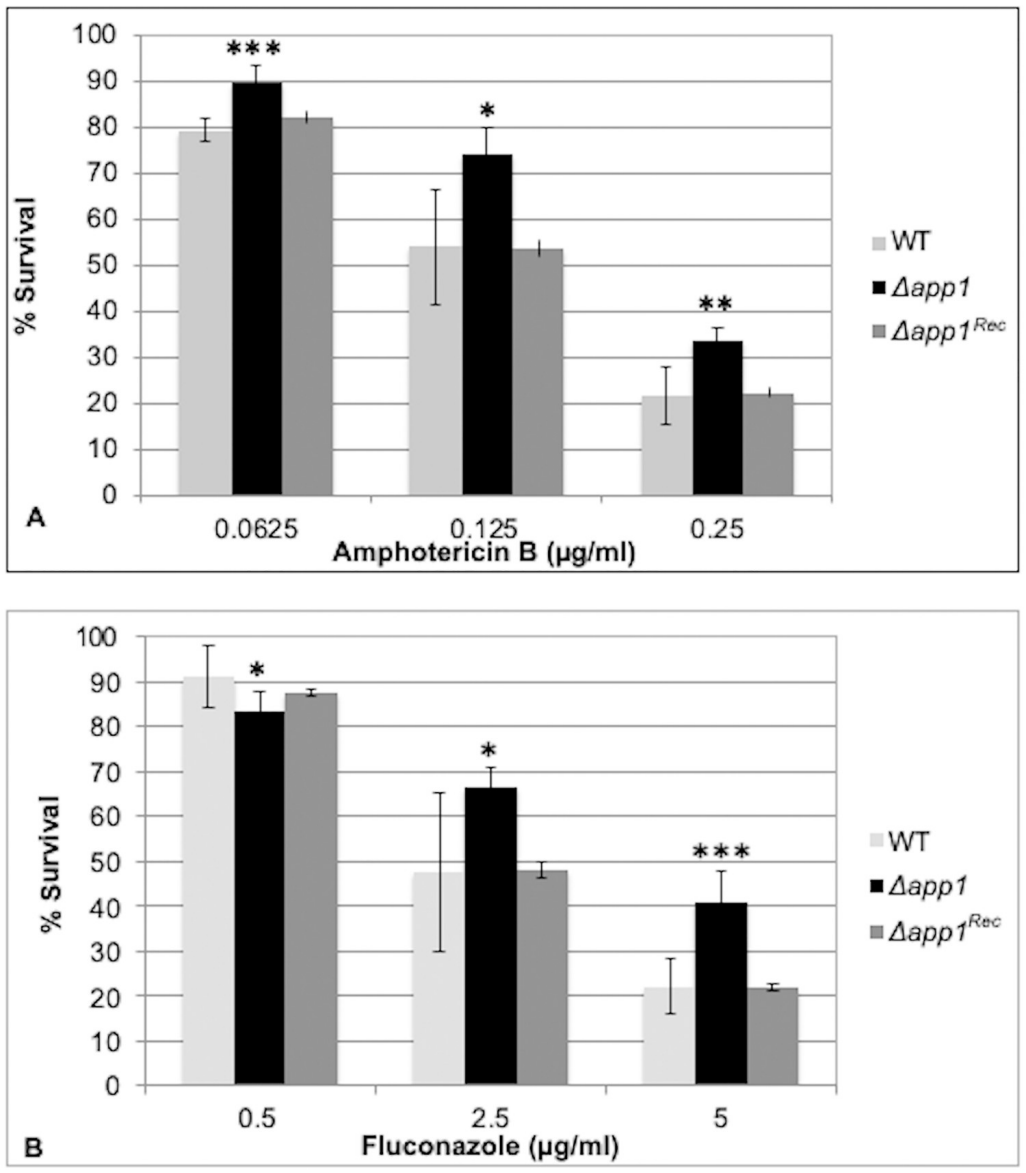
App1 increases the susceptibility of *C*. *neoformans* to antifungal drugs. *C*. *neoformans* (wild type (H99), *Δapp1*, or *Δapp1*^*Rec*^) was grown in YPD media with amphotericin B (A) or fluconazole (B) for 90 minutes and then plated on solid YPD to measure CFU after 2 days. Survival was measured by comparing treated cells to a nontreated control. Data show that the presence of App1 increases the susceptibility of *C*. *neoformans* to both amphotericin B and fluconazole. *p<0.05, **p<0.005, ***0<0.001 using student’s t-test. Data are averages +/- standard deviations for 5 or 6 separate experiments (n = 5 for amphotericin B and n = 6 for fluconazole).

Wild type or *Δapp1 C*. *neoformans* cells (10^5^) were treated with 0.4 μg/ml AMB (Fig 2A) or 3 μg/ml FLC (Fig 2B) and grown at 30°C for 46 hours while shaking. The OD 540 nm was measured every 2 hours to obtain growth curves. Control cultures were grown without treatment with an equal volume of DMSO for the FLC control or H_2_O for the AMB control, as these were the antifungal carrier solutions. Fig 2 shows that the presence of App1 in wild type cells leads to a longer lag phase when treated with AMB, suggesting App1 results in a slower recovery following treatment with AMB. *Δapp1* cells start the log phase and begin exponential growth approximately 34 hours after initial exposure to AMB but WT cells do not begin the log phase until approximately 38–40 hours after initial exposure to AMB. App1 did not affect growth following FLC treatment. Data are representative of at least 4 independent trials.

### App1 increases the susceptibility of *C*. *neoformans* to amphotericin B and fluconazole

A killing assay was done to determine whether App1 affects the susceptibility of *C*. *neoformans* to AMB or FLC. Cell cultures of either H99 (wild type), *Δapp1*, or *Δapp1*^*Rec*^ were treated with increasing doses of the drug for 90 minutes and then plated to measure % survival. Survival was then compared between H99 and *Δapp1* to determine whether the presence of App1 affects the killing of *C*. *neoformans* by the drugs. *Δapp1*^*Rec*^ was used as a control to ensure any differences observed between H99 and *Δapp1* were due to the presence of App1 and not due to defects from the process of making the *Δapp1* strain. Fig 3 shows the results of this killing assay by presenting % survival of the three strains at each concentration. Fig 3a shows that when treated with AMB, *Δapp1* cells have higher survival rates compared to WT cells. This difference was significant at all three concentrations of AMB tested: 0.0625, 0.125, and 0.25 μg/mL. (N = 5, *p<0.05 for 0.125 μg/mL, **p<0.005 for 0.25 μg/mL, and ***p<0.001 for 0.0625 μg/mL, student t test). Similar results are shown in Fig 3b, showing the effect App1 has on the survival of *C*. *neoformans* following treatment with FLC. Survival rates are higher in *Δapp1* cells compared to WT cells at concentrations of 2.5 and 5 μg/mL, suggesting that App1 increases the susceptibility of *C*. *neoformans* to FLC. WT cells had a higher survival at the lowest concentration of 0.5 μg/mL. These findings were found to be significant at all three concentrations tested. (N = 6, *p<0.05 for 0.5 and 2.5 μg/mL and ***p<0.001 for 5 μg/mL, student t test). The *Δapp1*^*Rec*^ strain shows similar results as the H99 strain, suggesting that any differences in the *Δapp1* strain are due to the absence of App1 and are not artifacts due to the process of making the *Δapp1* strain.

## Discussion

Cryptococcosis is a significant worldwide health burden, causing meningioencephalitis primarily in immunocompromised individuals. The yeast produces multiple virulence factors that aid in its pathogenicity. The crucial initial interaction of this fungal pathogen with the host immune response can be affected by App1, a virulence factor of *C*. *neoformans*. In this study, we present findings examining the relationship between App1 and the commonly used antifungal drugs AMB and FLC. We show here that *APP1* is downregulated by AMB but upregulated by FLC. The differing effects on *APP1* caused by these two antifungal drugs are surprising, as both drugs affect ergosterol in the fungal membrane. Microarray analysis shows FLC affects the expression of many *C*. *neoformans* genes [16]. App1 has been shown to be localized in the capsule and/or cell wall of *C*. *neoformans* [11]. Genes involved in membrane synthesis or cell wall maintenance were previously shown to be affected by FLC, possibly due to remodeling of the membrane following ergosterol disruption. Due to the effect of FLC on other genes involved in the cell wall, the upregulation of *APP1* we show here aligns with the localization of App1 in the cell wall or capsule of the yeast. Also, previous studies have shown AMB decreases capsule size [21]. Since App1 is localized in the cell wall/capsule, a decrease in *APP1* expression following AMB exposure aligns with these previous findings. Studies have also shown an increase in phagocytosis of CN after treatment with AMB. It was hypothesized this may be due to the decrease in capsule size [21]. Our findings align with this previous study and we hypothesize that a decrease in *APP1* expression may contribute to the mechanisms by which AMB leads to an increase in phagocytosis. In addition, App1 may have unknown functions in addition to its antiphagocytic properties. App1 may have roles in stress response pathways and may therefore be affected by exposure to antifungal drugs. Future studies can determine the mechanisms behind the effect of AMB and FLC on *APP1* expression, specifically examining how the different targets of these drugs leads to different effects on *APP1* expression. In addition, other potential functions of App1 can be examined.

Further, we show here that App1 increases the susceptibility of *C*. *neoformans* to both AMB and FLC and also delays the recovery of growth following AMB treatment. Follow up studies may determine the specific mechanism responsible for the role App1 has in the susceptibility of the yeast to these antifungal drugs. We hypothesize that App1’s effect on susceptibility to AMB and FLC may be a result of an increase in the intake of these drugs into the cells, a decrease of their efflux, or an increase in their conversion to active molecules. Potential proteins that App1 may interact with include Erg11, Afr1, Afr2, Mdr1, and Pdr11 [22–26]. Erg11 is the enzyme in the ergosterol synthesis pathway that FLC targets. Afr1, Afr2, Mdr1, and Pdr11 are efflux pumps that serve as transporters. App1 may potentially modulate expression or function of these efflux proteins. Measuring the levels of the antifungal drugs inside the cell in wild type compared to *Δapp1* cells will show whether App1 affects the intake or efflux of the drug. Studies can then be done to determine the mechanism behind any findings. Other genes have been found to be involved in the resistance of *C*. *neoformans* to antifungal drugs and are up or down regulated in response to antifungal exposure. App1 appears to follow this pattern and the data found here may lead to new information on the roles and functions of App1. It is interesting to note that at a FLC concentration of 0.5 μg/mL, App1 seems to have an opposite effect and decreases the susceptibility of cells to FLC. This suggests, the mechanism App1 uses to increase the susceptibility of cells to FLC requires concentrations above 0.5 μg/mL.

Additional future studies can be done testing the effect of FLC and AMB in combination on *APP1* expression since these drugs are usually given together during cryptococcal treatment. A limitation of the current work is that while we show a change in *APP1* mRNA levels following exposure to antifungal drugs, we have not shown the correlating protein levels. However, we expect the protein levels to change in the same pattern as seen with mRNA levels due to previous work showing both transcriptional and translational changes in *APP1* expression following environmental changes or stress [15].

Due to the role of App1 in the initial stages of cryptococcal disease and the differing effects of App1 depending on the immune state of the host, elucidating the mechanisms that control its expression as well as how it affects susceptibility to antifungal drugs is important in determining the most effective treatment per patient. Treatment of cryptococcosis is often individualized based on the immune impairment of the patient [27]. This could be especially important in the case of *APP1* expression, as the expression of *APP1* leads to a slower disease progression in immunocompromised hosts. The relationship between antifungal drugs and App1 is intricate. The affect FLC and AMB have on *APP1* expression may have impacts on a patient’s response to treatment. An upregulation of *APP1*, following FLC treatment, would lead to a decrease in phagocytosis, which would be beneficial in an immunocompromised host but disadvantageous for an immunocompetent host. The upregulation of *APP1* would also be beneficial to patients due to our finding that App1 increases the susceptibility of *C*. *neoformans* to antifungal drugs. A decrease in *APP1* expression could be harmful for an immunocompromised host, as an increase in phagocytosis can lead to faster dissemination of disease. In addition, a decrease in *APP1* expression may negatively affect response to treatment, as we show here that App1 increases the susceptibility of *C*. *neoformans* to antifungal drugs. This intricate relationship between App1 and antifungal drugs requires more examination to determine how it may play a role in choosing the best treatment plan per patient. Elucidating the relationship between virulence factors and antifungal drugs can lead to better treatments and specialized treatment plans.
